# Differential gene regulatory network analysis reveals transcriptional disruption in opioid

**DOI:** 10.1093/nargab/lqag031

**Published:** 2026-03-27

**Authors:** Jianlan Ren, Xu Wang, Feiyang Luan, Yeqing Chen, Le Gao, Wenzhe Ho, Zhi Wei

**Affiliations:** Department of Computer Science, New Jersey Institute of Technology, Newark, New Jersey, 07102, United States; Department of Pathology and Laboratory Medicine, Temple University Lewis Katz School of Medicine, Philadelphia, Pennsylvania, 19140, United States; Department of Computer Science, New Jersey Institute of Technology, Newark, New Jersey, 07102, United States; Department of Computer Science, New Jersey Institute of Technology, Newark, New Jersey, 07102, United States; Department of Computer Science, New Jersey Institute of Technology, Newark, New Jersey, 07102, United States; Department of Pathology and Laboratory Medicine, Temple University Lewis Katz School of Medicine, Philadelphia, Pennsylvania, 19140, United States; Department of Computer Science, New Jersey Institute of Technology, Newark, New Jersey, 07102, United States

## Abstract

Gene regulatory networks (GRNs) inferred from single-cell data offer a powerful lens for dissecting transcriptional regulation across biological conditions. Yet, statistical methods for comparing TF-level binary regulatory matrices—where edges represent the presence or absence of regulation—remain underdeveloped. Here, we introduce and benchmark five complementary statistical tests for group-level comparison of TF-level binary regulatory matrices. These include three global methods—a U-statistic-based dissimilarity test (Global_U), a distance-based pseudo-$F$ test (Global_F), and a PCA-based test—as well as two feature-level approaches: a per-feature U-test (Local_U) and Fisher’s exact test. Through extensive simulations spanning sparse, coordinated, balanced, and noisy signal structures, we show that global methods consistently outperform in detecting distributed regulatory shifts, particularly under correlation or noise. Applying this framework to single-nucleus RNA-seq data from human brain donors, we uncover astrocyte-specific regulatory alterations linked to opioid exposure. While each method captures distinct signal types, our results underscore the value of combining global and local tests to enhance sensitivity and interpretability. This unified framework provides a robust statistical foundation for GRN-based comparisons in single-cell studies.

## Introduction

Transcription factor (TF) regulatory networks orchestrate gene expression programs that underlie cell identity, development, and disease progression. Advances in single-cell and single-nucleus RNA sequencing (sc/snRNA-seq) have enabled the inference of putative gene regulatory networks (GRNs) at unprecedented resolution, facilitating the identification of cell-type-specific transcriptional mechanisms [1–3]. Tools such as CellOracle [2] and SCENIC [3] infer sample- or cell-type-specific TF–target gene (TG) links by integrating expression data with prior regulatory knowledge, producing binary matrices that encode the presence or absence of regulation [2, 3].

However, downstream statistical analysis of such GRNs—particularly when encoded as sparse, high-dimensional binary matrices—presents unique methodological challenges [4]. In many experimental settings, researchers seek to determine whether GRN activity differs between two biological conditions, such as disease versus control or treated versus untreated samples [5]. A common approach involves computing sample-specific GRNs and comparing the frequency of TF–TG regulatory links across groups [6–8]. Yet, traditional differential expression (DE) tools are ill-suited for such binary data. Feature-wise tests (e.g. Fisher’s exact test [9], Mann–Whitney U test [10]) often lack power when signals are sparse, diffuse, or coordinated across multiple genes.

Existing methods for differential network analysis typically focus on local dependency or correlation structures. For example, Zhang *et al. *[11] proposed a differential dependency network framework that identifies gene–gene interactions with condition-specific dependencies using Lasso-regularized conditional models and permutation tests. However, such approaches operate at the edge level and may miss global or distributed network changes. Similarly, DiffCoEx [12] builds on the WGCNA framework to identify differentially co-expressed modules by analyzing changes in gene–gene correlation patterns, but is primarily designed for continuous-valued expression matrices. In the single-cell setting, scTenifoldNet [13] constructs and compares GRNs using low-rank tensor decomposition and manifold alignment; while powerful, it is optimized for identifying pathway-level perturbations rather than testing global differences between binary networks. A recent review by Ideker and Krogan [14] emphasized the importance of differential network biology but highlighted the need for scalable statistical frameworks tailored to sparse or high-dimensional representations.

To address this gap, we propose an alternative nonparametric framework for comparing groups based on data-adaptive U-statistics. Inspired by the work of Wei and colleagues on U–statistics-based tests for multiple genes in genetic association studies [15], we developed a global U–statistics test (Global_U) that measures within- and between-group dissimilarity using a Hamming kernel. The classical U–statistic formulation [16] provides a flexible, distribution-free approach to compare pairwise similarities or distances across groups while accommodating high-dimensional settings. Conceptually, our method aligns with earlier score-based similarity tests developed for haplotype association [17], but is tailored to sparse binary matrices derived from GRNs.

We further adapted the distance-based pseudo-$F$ test (Global_F) from PERMANOVA [18], which was originally designed to test differences between multivariate sample centroids in ecological data. In its original form, PERMANOVA partitions sums of squares of pairwise distances to calculate an $F$-ratio and evaluates significance through permutations. Here, we generalize this approach to binary regulatory networks by defining dissimilarity through Jaccard distances. For context, the $F$-test itself, as classically described by Duncan [19], compares variance components to test hypotheses about group means. In our adaptation, the pseudo-$F$ statistic operates in a distance-based framework rather than relying on parametric assumptions about variance homogeneity.

In addition to these two global methods, our framework also incorporates a principal component analysis (PCA)-based test, which leverages dimension reduction to assess separation between groups in a low-dimensional space. These three global approaches are complemented by two feature-level tests: a per-feature U–test (Local_U) and a Fisher’s exact test. Together, these five methods provide a flexible toolkit for detecting group-level differences in TF-level binary regulatory matrices.

Opioid use disorder (OUD) is a chronic relapsing condition characterized by persistent neurobiological adaptations in reward, stress, and cognitive control circuits. Beyond neuronal dysfunction, growing evidence implicates glial cells—particularly astrocytes—in modulating synaptic plasticity, neuroinflammation, and homeostatic responses to chronic opioid exposure. However, the regulatory programs underlying astrocyte-specific transcriptional remodeling in OUD remain poorly characterized, motivating the need for network-level approaches that go beyond DE analyses.

The rest of this paper is organized as follows. In the Statistical Methods section, we describe five complementary approaches for comparing group-level patterns in high-dimensional binary regulatory networks, including the global U-statistic, distance-based pseudo-$F$, PCA-based separation, and two feature-level tests. We then illustrate the properties of these methods through extensive simulations under diverse network signal structures. Finally, we demonstrate their utility by applying them to biological case studies, followed by a discussion of findings and limitations.

## Materials and methods

### Main methods

We analyzed binary TF regulatory networks, represented as a matrix $X \in \lbrace 0,1\rbrace ^{G \times N}$, where each entry $x_{ij}$ indicates whether gene $i$ is active (1) or inactive (0) in sample $j$. Samples were divided into two groups (e.g. control versus treatment), and our objective was to test for overall differences in regulatory activity patterns between these groups. Rather than relying solely on per-feature comparisons, we developed and benchmarked five complementary statistical approaches, including three global tests and two feature-level tests.

#### Global U-statistic

We implemented a nonparametric U-statistics-based test [16] to quantify between-group dissimilarity relative to within-group variability [15], using a Hamming distance kernel [20]:


\begin{eqnarray*}
\phi (X_i, X_j) = \sum _{g=1}^G \mathbb {I}(X_{ig} \ne X_{jg}),
\end{eqnarray*}

where $X_i$ and $X_j$ are binary vectors for two samples. Average pairwise distances were computed within each group ($U_{AA}$ and $U_{BB}$) and between groups ($U_{AB}$), and a dissimilarity ratio was formed:


\begin{eqnarray*}
T_d = \frac{B}{W},
\end{eqnarray*}

with $B$ and $W$ denoting between- and within-group contributions, respectively. Statistical significance was assessed by permuting group labels to generate an empirical null distribution of $T_d$.

#### Global F-statistic

We adapted a distance-based pseudo-$F$ test, similar in spirit to PERMANOVA [18], using Jaccard distances [21] between sample vectors. The test statistic,


\begin{eqnarray*}
F^{*} = \frac{D_{\mathrm{between}}}{D_{\mathrm{within}}},
\end{eqnarray*}

compares average between-group dissimilarity with average within-group dissimilarity. Significance was evaluated by permuting group labels to obtain the null distribution of $F^{*}$. Here, the Global U test uses Hamming distance to capture absolute edge-wise mismatches across networks, whereas the Global F test uses Jaccard distance to focus on differences in overlapping active edges. These two distance measures therefore provide complementary sensitivity for sparse TF-level binary regulatory matrices.

#### PCA-based group separation

For each TF-level binary target-by-sample matrix, we performed PCA to obtain a low-dimensional representation of samples [22]. Let $z_i$ denote the projection of sample $i$ onto the first principal component. Group separation was quantified by the absolute difference in mean PC1 scores between case and control samples,


\begin{eqnarray*}
S = \left| \bar{z}_{\mathrm{Case}} - \bar{z}_{\mathrm{Control}} \right|.
\end{eqnarray*}

Statistical significance was assessed via permutation testing by randomly permuting group labels and recomputing $S$ to obtain an empirical null distribution.

#### Fisher’s exact test

As a baseline global test, we aggregated the binary gene–sample matrix across all features to obtain the total number of “active” entries within each group. Let $X \in \lbrace 0,1\rbrace ^{n \times G}$ denote the binary matrix with $n$ samples and $G$ genes, and let $\mathcal {A},\mathcal {B}$ denote the index sets of Group A and Group B, respectively, with $|\mathcal {A}|=n_A$ and $|\mathcal {B}|=n_B$. We define


\begin{eqnarray*}
O_A = \sum _{i \in \mathcal {A}} \sum _{g=1}^G X_{ig}, \qquad O_B = \sum _{i \in \mathcal {B}} \sum _{g=1}^G X_{ig},
\end{eqnarray*}

as the observed numbers of active entries in Groups A and B. The corresponding numbers of inactive entries are


\begin{eqnarray*}
Z_A = n_A G - O_A, \qquad Z_B = n_B G - O_B.
\end{eqnarray*}

These counts form a $2 \times 2$ contingency table


\begin{eqnarray*}
\begin{array}{c|cc}& \mathrm{Active} & \mathrm{Inactive} \\\hline \text{Group A} & O_A & Z_A \\\text{Group B} & O_B & Z_B \end{array}
\end{eqnarray*}

on which Fisher’s exact test [23] was applied to assess differences in overall regulatory burden between groups. To account for potential dependence and small-sample discreteness, we further obtained empirical $P$-values, $P_{\mathrm{perm}}$ , by randomly permuting group labels $R$ times and recalculating the Fisher statistic to form a permutation null distribution:


\begin{eqnarray*}
P_{\mathrm{perm}} = \frac{1 + \sum _{r=1}^R \mathbf {1}\lbrace P^{(r)} \le P^{\mathrm{obs}}\rbrace }{R+1},
\end{eqnarray*}

where $P^{\mathrm{obs}}$ is the observed Fisher $P$-value and $P^{(r)}$ is the Fisher $P$-value under the $r$th permutation.

#### Local U-test

To assess feature-level differences between groups, we applied the Mann–Whitney U-test [10] independently to each gene. For gene $i \in \lbrace 1,\dots ,G\rbrace$, let $P_i$ denote the resulting two-sided $P$-value. These per-gene $P$-values were then aggregated using Fisher’s method,


\begin{eqnarray*}
T = -2 \sum _{i=1}^G \ln (P_i),
\end{eqnarray*}

which under the assumption of independence follows a $\chi ^2$ distribution with $2G$ degrees of freedom. To account for correlation among genes, we assessed significance via label permutation: group labels were permuted $R$ times, per-gene U-tests were recomputed to obtain $\lbrace P_i^{(r)}\rbrace$, and the combined statistic $T^{(r)}$ was recalculated for each replicate. The empirical $P$-value was then obtained as


\begin{eqnarray*}
P_{\mathrm{perm}} = \frac{1 + \sum _{r=1}^R \mathbf {1}\lbrace T^{(r)} \ge T^{\mathrm{obs}}\rbrace }{R+1},
\end{eqnarray*}

where $T^{\mathrm{obs}}$ is the observed Fisher combined statistic. This approach leverages per-gene rank-based comparisons while providing a global test that is robust to heterogeneous or bidirectional effects across genes.

Together, these five approaches provide complementary perspectives on detecting group-level differences in TF-level binary regulatory matrices. Global methods such as Global_U and Global_F are designed to capture coordinated or distributed shifts across multiple features, while feature-level tests, such as Fisher’s exact test or Local_U, offer fine-grained resolution for detecting localized changes. However, feature-level approaches may suffer from reduced power in settings with bidirectional or symmetric signal distributions, such as the balanced simulation scenario.

### Simulation design

To benchmark method performance across a range of biologically and technically relevant scenarios, we constructed a suite of simulation settings that generate binary gene-by-sample matrices $X \in \lbrace 0,1\rbrace ^{G \times N}$, where $G = 80$ features (e.g. TF–TG links) and $N = 40$ samples are evenly split into two groups. Each simulation is parameterized by a signal fraction $\gamma \in \lbrace 0.05, 0.10, 0.15, 0.20, 0.25, 0.30\rbrace$, indicating the proportion of genes that carry group-specific signal. The remaining features follow a background noise model with low activation probability. We evaluated all methods under the following five scenarios:

#### Normal signal

This baseline setting models sparse, unidirectional regulatory activation, where only a small subset of features (5%–30%) are differentially active between groups. Signal features are activated with high probability ($P_\mathrm{ A} = 0.3$) in Group A and low probability ($P_\mathrm{ B} = 0.05$) in Group B. The remaining genes follow a common background model ($P = 0.1$) with no group difference. This structure reflects realistic biological scenarios where a limited number of TF targets are upregulated in one condition—such as disease-specific transcriptional activation or pathway-specific stimulation—while most features remain inactive or randomly active. In our real dataset, only **1 out of 160 TFs (0.6%)** were classified as Normal, likely due to the rarity of clear, unidirectional signal without technical noise or symmetric regulation in practice.

#### Balanced signal

This scenario models symmetric but group-specific regulation. As in the Normal setting, a small fraction of features are designated as signal genes, but here the signal is bidirectional: half are activated in Group A ($P_\mathrm{ A} = 0.3$, $P_\mathrm{ B} = 0.05$), and the other half in Group B ($P_\mathrm{ A} = 0.05$, $P_\mathrm{ B} = 0.3$). The rest follow the background model. This setting simulates conditions where both groups exhibit distinct but non-overlapping regulatory programs, such as in immune versus metabolic activation across disease subtypes. In our dataset, this pattern is remarkably prevalent, with **120 out of 160 TFs (75.0%)** classified as Balanced, suggesting widespread, symmetric regulatory differences across conditions.

#### Noisy signal

To model technical artifacts such as measurement error or batch effects, we add unstructured binary noise to the Normal signal matrix. Specifically, random Bernoulli noise ($P = 0.05$) is added independently to each matrix entry, simulating spurious activation events not associated with biological groups. This leads to widespread contamination of true signal and background, reflecting low-quality or heterogeneous experimental datasets. In our real data, **17 out of 160 TFs (10.6%)** showed this Noisy pattern, indicating that technical noise or high baseline activation may obscure clear group-level signals in a moderate proportion of TFs.

#### Coordinated signal

This scenario captures biologically meaningful coregulation by introducing correlated activation among signal features in one group. A shared binary activation pattern is applied across all signal genes in Group A, simulating modular regulation by a common TF or pathway. Group B remains governed by independent Bernoulli noise. This reflects realistic gene programs such as co-expression within pathways or modules. In our dataset, **4 out of 160 TFs (2.5%)** were labeled as Coordinated, which may reflect the scarcity of perfectly coregulated modules among individual TF targets in sparse, binary data.

#### Null signal

In the Null setting, all genes are sampled from a common Bernoulli distribution ($P = 0.1$), with no group-specific structure. This simulates data under the global null hypothesis and provides a benchmark for type I error control. In real data, **18 out of 160 TFs (11.3%)** were classified as Null, likely reflecting TFs with low or random activity across all samples, lacking biological or technical signal.

Each simulation was repeated 500 times per configuration and evaluated under three significance thresholds ($\alpha \in \lbrace 0.05, 0.01, 0.005\rbrace$) using $B = 2000$ permutations per test. Signal features were randomly permuted within each matrix to avoid position bias. The simulated matrices were transposed ($N \times G$) to reflect sample-wise comparisons in all downstream tests.

### Application to real datasets

To test method performance on biological data, we applied the framework to snRNA-seq data from the SCORCH program. Using CellOracle [2], we constructed sample-specific GRNs from promoter-based TF–target priors. Regulatory links were defined as active if the TF–TG Pearson correlation had a Benjamini–Hochberg-adjusted $P \le 0.05$, resulting in one binary matrix per TF with TGs as rows and samples as columns.

For clarity, per-donor TF-level binary regulatory matrices were constructed as follows: (i) astrocyte nuclei were identified based on cell-type annotations from the original SCORCH snRNA-seq preprocessing; (ii) astrocyte-specific expression matrices were extracted for each donor; (iii) CellOracle was applied using promoter-based TF–TG priors to define candidate regulatory edges; (iv) for each TF–TG pair, correlations between TF and TG expression across astrocyte nuclei within the same donor were computed; (v) *P*-values were adjusted using the Benjamini–Hochberg procedure; and (vi) TF–TG edges with adjusted $P \le 0.05$ were retained and encoded as binary (1 = active, 0 = inactive), yielding one TF-level binary target-by-sample matrix per donor.

After standard quality control and filtering, the astrocyte expression matrix retained ~18 000 genes per donor for downstream analysis. Cell types were identified using established marker genes and annotations provided by the original SCORCH study.

#### Classification of real data into simulation categories

To contextualize simulation settings within real-world data, we classified each TF–TG binary matrix from the astrocyte dataset into one of five simulation-inspired categories: *Normal, Coordinated, Balanced, Noisy*, and *Null*. For each TF, we computed key summary statistics: group-specific activation rates, signal asymmetry, technical noise level (estimated as off-target activation), and within-group correlation among signal TGs.

Classification rules mirrored the structural assumptions of our simulations. For instance, matrices with moderate asymmetry ($>0.05$) and low noise/correlation were labeled *Normal*, while those with high within-group correlation and moderate activation were marked as *Coordinated*. TFs with bidirectional, symmetric activation patterns were labeled *Balanced*, and matrices with minimal group differences and high background activity were labeled *Noisy* or *Null* depending on signal sparsity.

This mapping allowed us to validate that our simulation settings reflected patterns observed in real data. Notably, most TFs clustered into the *Balanced* or *Noisy* categories, with fewer exhibiting clean *Normal* or *Coordinated* signals—highlighting the complexity and heterogeneity of real transcriptional regulation.

#### Statistical testing on TF activity matrices

Each TF’s binary activation matrix was tested for group differences using our suite of methods: Global_U, Global_F, Local_U, PCA-based score, and Fisher’s test. Significance was evaluated via permutation testing and adjusted for multiple comparisons (BH correction) [24].

#### Effect size estimation

To quantify TF-level effects, we summarized group-wise differences in target activation using the mean of the top-$k$ absolute activation differences:


\begin{eqnarray*}
\text{Effect size}_{\mathrm{TF}} = \frac{1}{k} \sum _{i=1}^{k} \left| \mathrm{activation}_{\mathrm{Case}, i} - \mathrm{activation}_{\mathrm{Control}, i} \right|,
\end{eqnarray*}

where $k = 10$ in practice, and targets are ranked by absolute activation difference. If fewer than $k$ targets were available for a given TF, all targets were used.

This choice reflects the sparse nature of TF–target regulatory programs, in which a TF typically exerts strong regulatory effects on a limited subset of its targets. Averaging over all targets would dilute meaningful signal with a large number of inactive or weakly changing edges, while focusing on a single maximum difference would be overly sensitive to noise. Using a small top-$k$ therefore provides a stable and interpretable summary of TF-specific regulatory disruption. As shown in Supplementary Table S2, TF effect size rankings were highly concordant across values of $k$, indicating that the main conclusions are not driven by a specific choice of $k$.

#### Visualization and interpretation

Volcano plots were generated for each method, with effect size on the $x$-axis and $-\log _{10}\ (\text{adjusted } P)$ on the $y$-axis. TFs passing a BH-adjusted threshold of $P < 0.1$ were labeled, and plots were created using R (version 4.4.1) [25].

To assess cross-method consistency, we constructed an UpSet plot by converting results into a binary TF-by-method matrix indicating significance ($P_{\mathrm{adj}} < 0.1$). This visualization revealed overlapping and unique TFs detected across tests.

#### Pathway enrichment

To interpret the functional consequences of differential TF activity, we performed preranked gene set enrichment analysis (GSEA) using Reactome pathways [26, 27]. For each TG, we defined a group-level differential activation score as the difference in mean binary activation between case and control samples,


\begin{eqnarray*}
D_g = \bar{x}_{g,\mathrm{Case}} - \bar{x}_{g,\mathrm{Control}},
\end{eqnarray*}

where $\bar{x}_{g,\cdot }$ denotes the average binary activation of gene $g$ within each group. Genes were ranked by $D_g$, and enrichment analysis was performed using the standard GSEA framework. Pathways with false discovery rate $q \le 0.05$ were retained, and leading-edge subsets were used for downstream interpretation.

#### Computational runtime

On the SCORCH astrocyte dataset, the full TF-level analysis across all methods completed in ~2–3 h on a standard workstation (single CPU core). Runtime was primarily driven by the number of TFs evaluated and permutation-based procedures used in global tests, while Local U and Fisher’s exact test exhibited comparable per-TF runtimes. Analyses were not optimized for parallelization.

## Results

### Simulation studies

**Figure 1.**
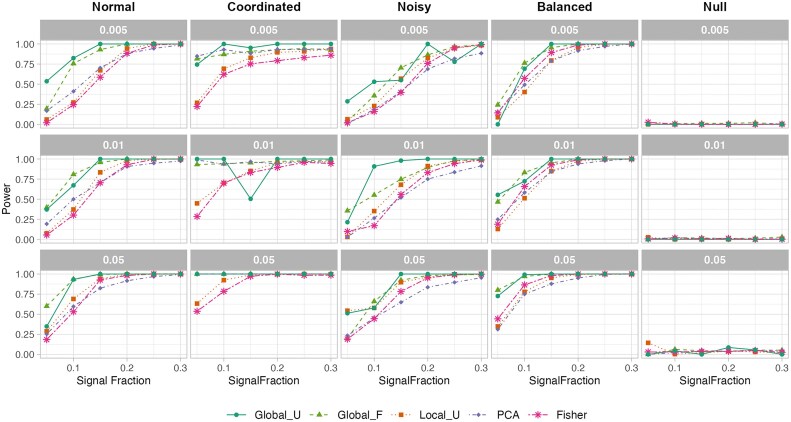
Statistical power of five methods across simulations. Columns correspond to simulation settings (Normal, Coordinated, Noisy, Balanced, and Null); rows indicate different significance thresholds ($\alpha = 0.005, 0.010, 0.015$). Each curve shows the proportion of detections (power) as a function of signal fraction. Fisher refers to *Fisher’s exact test*.

We evaluated the performance of five statistical methods for detecting group-level differences in high-dimensional binary matrices across five simulation scenarios, each designed to probe distinct structural characteristics of the underlying signal. These methods included the global U-statistic (Global_U), distance-based pseudo-F (Global_F), PCA-score test (PCA), per-feature U-test (Local_U), and Fisher’s exact test. Figure 1 summarizes empirical power across methods and signal fractions under significance thresholds $\alpha \in \lbrace 0.05, 0.01, 0.005\rbrace$.

#### Normal signal

In the Normal signal setting—characterized by unidirectional activation of a small subset of regulatory links in one group—all methods showed increasing power with higher signal fractions. Both Global_U and Global_F consistently outperformed the others, achieving near-perfect power ($>0.95$) at signal fraction 0.25 and above across all significance levels. At the lowest signal fraction (0.05), Global_F attained 60% power at $\alpha = 0.05$, while Global_U reached 42%. These global methods remained substantially more sensitive than feature-level tests.

Local_U showed moderate power gains, from 52% at 0.05 signal fraction to over 90% at higher levels, outperforming PCA and Fisher. The PCA-based test lagged behind, particularly at low signal levels (e.g. 25% power at 0.05), while Fisher’s exact test remained consistently underpowered, rarely exceeding 10% power at low signal fractions. These results highlight the superior sensitivity of global rank- and distance-based statistics in detecting weak but coherent regulatory activation patterns embedded within binary matrices.

#### Coordinated signal

In the Coordinated signal setting—where signal features in Group A are coregulated and exhibit high within-group correlation—all methods demonstrated strong power due to the structured activation pattern. Global_U and Global_F were again the top performers, each achieving 100% power across all signal fractions and $\alpha$ levels. Fisher’s exact test also benefited from the coherence of signals, reaching 99.6% power at signal fraction 0.05 and $\alpha =0.05$.

In contrast, Local_U was the weakest performer in this setting, starting at 75.2% power at the lowest signal level and reaching 99.8% only at the highest signal fraction. The PCA method showed intermediate performance—improving over the Normal setting—but still trailed the global tests. These findings reinforce that coordinated structure among signals strongly favors global, structure-aware methods over per-feature testing approaches.

#### Noisy signal

In the Noisy signal setting—where random binary noise is added across the matrix to simulate technical artifacts—all methods experienced a moderate drop in sensitivity, especially at lower signal fractions. However, the global methods remained relatively robust. Global_U and Global_F achieved the highest power across nearly all conditions, with both surpassing 0.9 power at signal fraction 0.25 and above. At the lowest signal fraction (0.05), Global_U retained stronger performance (64% at $\alpha = 0.05$) compared to Global_F (42%), highlighting the benefit of distance-based aggregation in the presence of noise.

Local_U and Fisher showed moderate resilience, achieving 54% and 27% power, respectively, at signal fraction 0.05 and improving to over 90% at higher fractions. In contrast, PCA exhibited the weakest performance among all methods in this setting, with power only reaching 15% at signal fraction 0.05 and remaining consistently lower than other methods across all thresholds. These findings emphasize that distance- and rank-based global statistics are better suited for noisy binary data, while dimension-reduction approaches such as PCA are more susceptible to noise contamination and signal dilution.

#### Balanced signal

In the Balanced signal setting, both groups exhibit high and symmetric activation probabilities ($P_\mathrm{ A} \approx P_\mathrm{ B} \approx 0.5$) for the same subset of signal features. This structure mimics coordinated or bidirectional regulation across conditions, where group differences are minimal or subtle. As expected, all methods exhibited relatively low statistical power in this setting, with limited ability to detect group-level differences despite the presence of high activity. Global_U and Global_F showed marginally better performance compared to others, achieving power values up to 0.46 and 0.42, respectively, at $\alpha =0.05$ when signal fraction reached 0.25. These global methods appear to pick up subtle distributional shifts or residual asymmetry, though their sensitivity remains modest in this near-null scenario.

Local_U and Fisher demonstrated similar low power profiles, with values ranging from 0.08 to 0.25 across signal levels, and never exceeding 0.3 even at higher signal fractions. The PCA-based test consistently underperformed all other methods, rarely exceeding 10% power, reflecting its limited utility under highly symmetric and nonseparable conditions. These results reinforce that Balanced settings, despite high feature activation, present a statistical challenge due to minimal group differences. Global methods offer slight advantages by capturing subtle distributional discrepancies, but overall power remains constrained.

#### Null signal

Under the null setting, where no group differences were present and all matrix entries were independently sampled from a Bernoulli distribution ($P = 0.1$), all methods controlled type I error rates reasonably well. Global_U, Global_F, and PCA showed no evidence of inflation, with empirical power remaining at or below the nominal $\alpha$ thresholds across all levels. Fisher’s exact test was slightly liberal but remained close to the expected type I error, yielding a power of 0.035 at $\alpha = 0.05$. Local_U exhibited marginal inflation, with a type I error rate of 0.043 at the same threshold. At more stringent $\alpha$ levels ($\alpha = 0.01$ and 0.005), all methods remained conservative. These findings confirm the reliability of the global and structure-aware tests under the null, while suggesting mild over-sensitivity of per-feature methods at nominal thresholds.

Overall, Global_U and Global_F demonstrated consistently strong and complementary performance across diverse signal structures. Global_U excelled at detecting unidirectional signals—especially at low signal fractions—and maintained robust performance under normal, noisy and correlated conditions. Global_F was particularly effective in scenarios with balanced activation pattern, reflecting its sensitivity to structured, bidirectional signals. The PCA-based test performed well in coordinated settings where global covariance structures were prominent, but showed limited power under normal, balanced or noisy conditions. Local_U achieved moderate power in noisy settings but suffered in balanced and coordinated scenarios. Fisher’s exact test remained consistently conservative and underpowered, especially in weakly structured signals. These results highlight the strengths of global, structure-aware methods—particularly Global_U—for detecting subtle yet coordinated regulatory differences in high-dimensional binary matrices. In contrast, conventional per-feature tests such as Local_U and Fisher often lack the sensitivity required to detect distributed signal patterns. A complete description of simulation setups is provided in the Additional Methods under Simulation Design.

### Transcription factor-level network disruption in OUD data set (astrocyte)

We analyzed TF network disruption associated with OUD using snRNA-seq data from the SCORCH program, comprising 95 postmortem brain donors (45 with documented opioid exposure and 50 matched controls) sampled from Michigan and Florida. After quality control, preprocessing, and normalization, we focused on astrocytes—the second most abundant cell type—yielding 31 984 high-quality nuclei.

Cell-type-specific GRNs were constructed for each sample using CellOracle with promoter-based TF–TG priors. This resulted in profiles for 119 TFs and 2274 predicted TGs. For each sample, TF–TG correlations were computed from astrocyte-specific expression profiles, and significant interactions were defined by an adjusted $P$-value ${\le} {0.05}$, yielding binary (1/0) regulatory matrices representing active TF–TG links.

**Figure 2.**
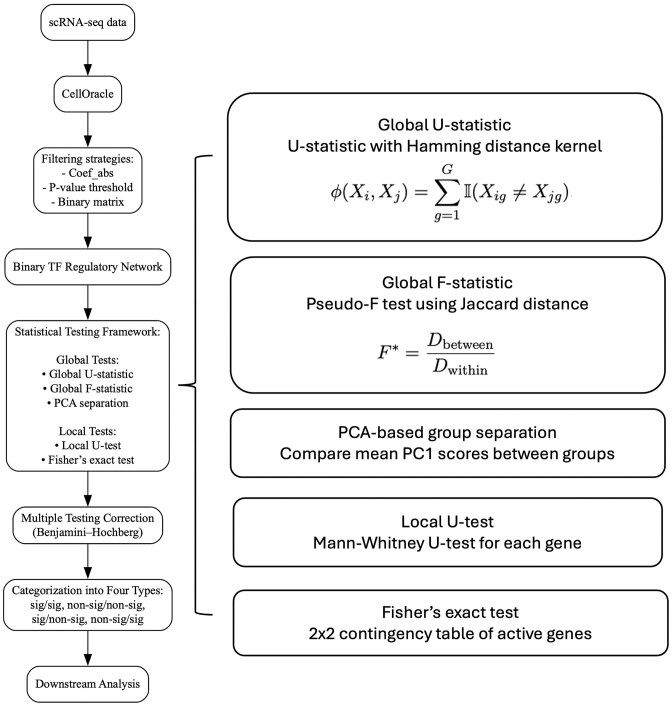
Analytical workflow for comparative network analysis. snRNA-seq data were processed to generate sample-specific binary TF regulatory networks using CellOracle. Filtering strategies based on effect size and significance thresholds produced binary matrices of active TF–TG interactions. Global tests (U-statistic, pseudo-$F$, PCA-based separation) and local tests (Mann–Whitney U, Fisher’s exact test) were applied to detect group-level regulatory differences. Multiple testing correction and categorization of significant TF–TG relationships by DE status enabled downstream mechanistic interpretation.

To assess group-level differences between OUD and control networks, we implemented a statistical framework integrating both global and local approaches (Fig. 2). Global tests included a U-statistic based on Hamming distance, a pseudo-$F$ test using Jaccard distances, and a PCA-based separation of group centroids. Local tests comprised per-feature Mann–Whitney U-tests and Fisher’s exact tests to detect differential activity at individual TF–TG links. Multiple testing correction was performed using the Benjamini–Hochberg procedure, and significant TFs were further stratified by the DE status of their TGs. This integrated framework enabled systematic identification of transcriptional regulatory disruptions in astrocytes associated with OUD.

#### Distinct and shared transcription factor discoveries across methods

**Figure 3.**
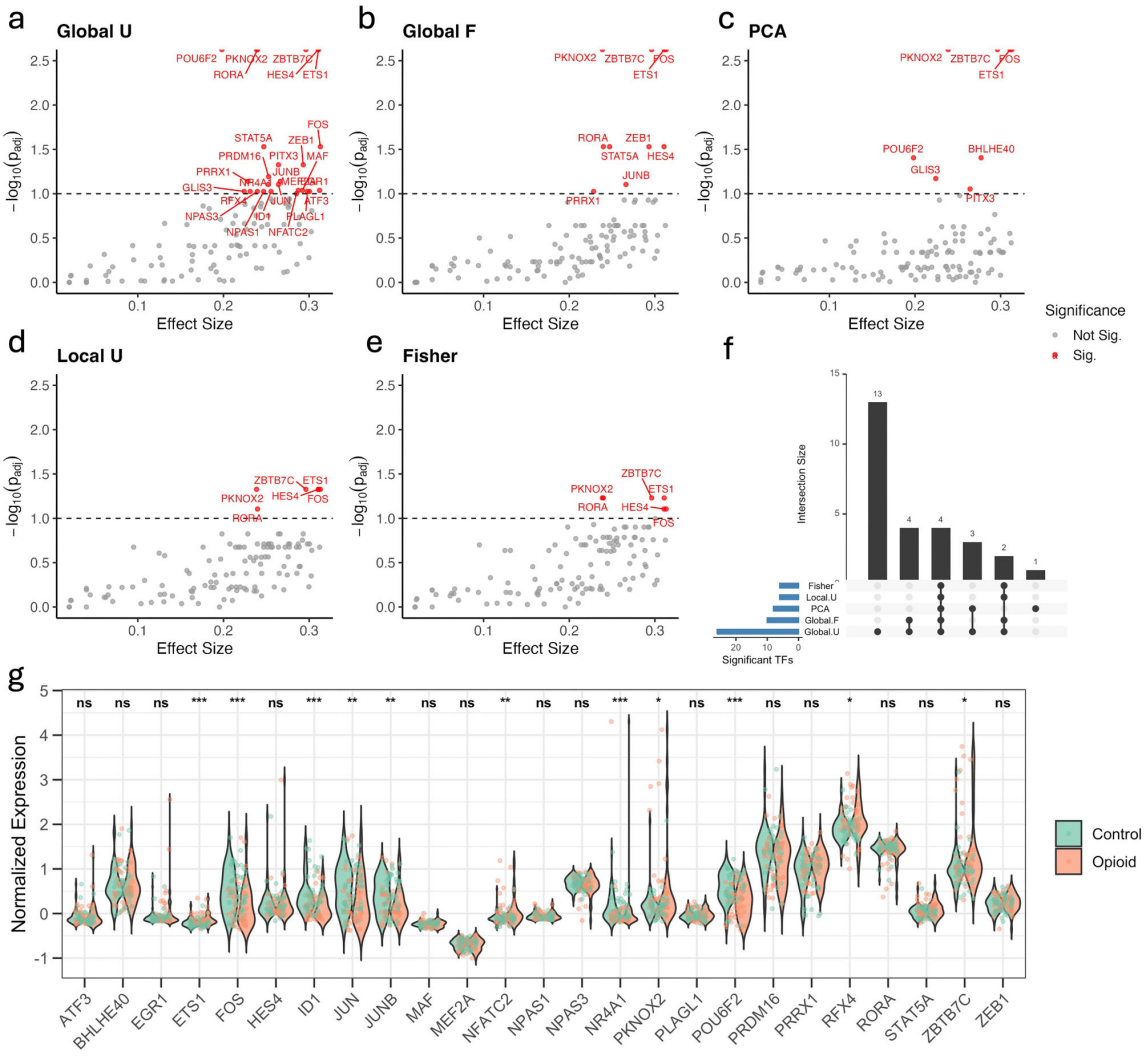
TF activity in astrocytes from SCORCH snRNA-seq data. (**a**–**e**) Volcano plots for five statistical methods (Global_U, Global_F, PCA score, Local_U, and Fisher’s exact test) display the relationship between effect size and $-\log _{10}$(adjusted $P$-value) for each TF. Significant TFs are highlighted. (**f**) UpSet plot of TFs identified by different statistical methods. Bar heights represent intersection sizes, and bar colors with connected dots indicate overlapping method sets. (**g**) Violin plots of TF expression show per-sample distributions grouped by category. Significance from the Mann–Whitney U test is indicated above: ***$P \le 0.001$, **$P \le 0.01$, *$P\le 0.05$, and ns (not significant).

These methods exhibited both overlap and complementarity in their discoveries. We visualized TF results using volcano plots for each method (Fig. 3a–e). Global_U uniquely identified 13 TFs, including *ATF3, EGR1, ID1, JUN, MAF, MEF2A, NFATC2, NPAS1, NPAS3, NR4A1, PLAGL1, PRDM16*, and *RFX4*. Four TFs, such as *JUNB, PRRX1, STAT5A*, and *ZEB1*, were shared between Global_F and Global_U. Four TFs—*ETS1, FOS, PKNOX2*, and *ZBTB7C*—were consistently identified across all methods. PCA also identified one unique TF (*BHLHE40*), while Global_U method shared *GLIS3, PITX3*, and *POU6F2* with PCA. Moreover, *HES4* and *RORA* are shared among all methods except PCA. The overlap across methods is illustrated in the UpSet plot (Fig. 3f). Notably, *FOS, ZBTB7C*, and *ETS1* demonstrated strong significance and large effect sizes under both Global U and Global F, highlighting them as potential regulatory drivers in opioid-related astrocyte reprogramming. For TF-level visualization and prioritization, we used a BH-adjusted significance threshold of 0.1, reflecting the exploratory nature of network-level analyses in sparse single-nucleus data; importantly, key TF discoveries were qualitatively stable under a more stringent threshold of 0.05 (Supplementary Table S1).

We further examined the expression levels of these TFs (Fig. 3g). Several, including *ATF3, BHLHE40, EGR1, HES4, MAF, MEF2A, NPAS1, NPAS3, PLAGL1, PRDM16, PRRX1, RORA, STAT5A*, and *ZEB1*, showed no significant gene expression differences (adjusted $P > 0.05$), highlighting the advantage of network-based approaches in detecting regulatory shifts beyond expression.

#### Functional enrichment and biological relevance of identified TFs

**Figure 4.**
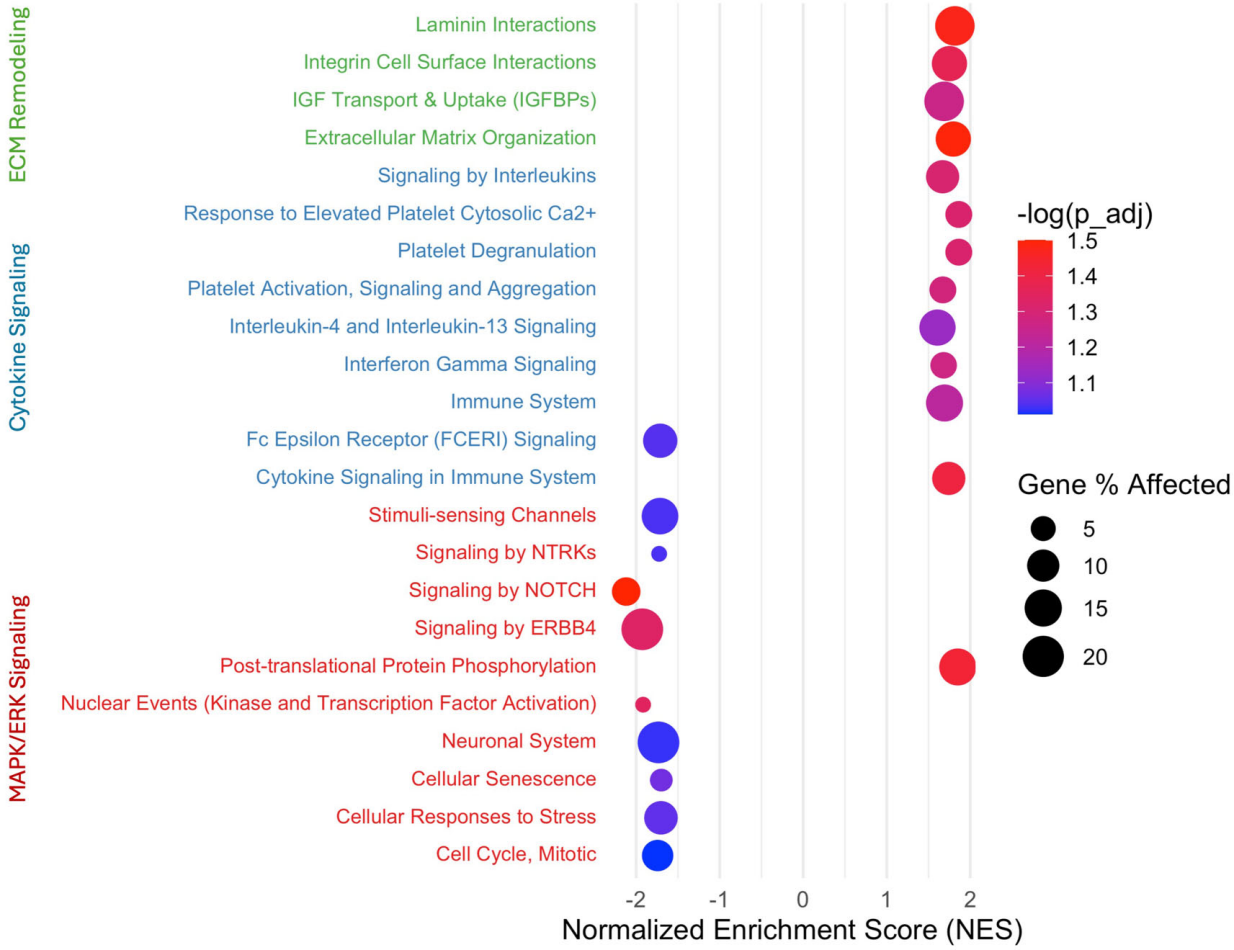
Enriched pathway associations for significant TFs of Astrocytes from SCORCH snRNA-seq data. Dot plot summarizing enriched pathway associations for significant TFs. Dot size corresponds to the number of overlapping genes; colors indicate method overlap category.

To assess whether the TFs identified in the preceding analysis correspond to meaningful biological processes, we performed GSEA on the top 2000 genes in the dataset, ranked by expression variability (Fig. 4). Among the 23 Reactome pathways with adjusted $P$-values below 0.1, 13 were upregulated and 10 were downregulated in OUD astrocytes. These pathways clustered into three major functional categories, each strongly connected to the TFs identified in the first subsection.

MAPK/ERK signaling pathways, driven by targets of JUN, FOS, and EGR1, were broadly downregulated, with the exception of post-translational protein phosphorylation. Enriched pathways in this category included Signaling by NOTCH, Signaling by ERBB4, Nuclear Events (Kinase and Transcription Factor Activation), Neuronal System, Cellular Senescence, Cellular Responses to Stress, and Cell Cycle Mitotic.

Cytokine signaling pathways, linked to STAT5A and JUNB, were predominantly upregulated, with the exception of FCERI signaling. Enriched pathways here included Signaling by Interleukins, Response to Elevated Platelet Cytosolic Ca2+, Platelet Degranulation, Platelet Activation and Aggregation, Interleukin-4 and Interleukin-13 Signaling, Interferon Gamma Signaling, and Cytokine Signaling in the Immune System.

Extracellular matrix (ECM) remodeling pathways, associated with EGR1 and ATF3, were consistently upregulated, including Laminin Interactions, Integrin Cell Surface Interactions, IGF Transport and Uptake (IGFBPs), and Extracellular Matrix Organization.

Among all significant pathways, those with the highest percentages of genes affected were Signaling by ERBB4 (20.56%), Neuronal System (20.56%), IGFBPs (17.75%), Laminin Interactions (17.65%), and Post-translational Protein Phosphorylation (14.73%). Based on normalized enrichment scores (NES), the top five upregulated pathways were Response to Elevated Platelet Cytosolic Ca2+ (NES = 1.862992), Platelet Degranulation (1.862992), Post-translational Protein Phosphorylation (1.850455), Laminin Interactions (1.816539), and Extracellular Matrix Organization (1.797311). The top five downregulated pathways were Signaling by NOTCH (NES = –2.119105), Signaling by ERBB4 (–1.926436), Nuclear Events (Kinase and Transcription Factor Activation; –1.916834), Cell Cycle Mitotic (–1.742872), and Neuronal System (–1.732497).

Notably, several TFs implicated in these pathways—such as EGR1, STAT5A, and ATF3—showed no significant DE (adjusted $p > 0.05$). This highlights the strength of the network-based framework and, in particular, the global methods, which were more effective in capturing regulatory disruptions. EGR1 and ATF3 were identified by the Global_F method, while STAT5A was detected by both Global_U and Global_F. By assessing disruptions in TF–TG links rather than relying solely on TF expression levels, our approach reveals regulatory alterations that would otherwise remain undetected, providing biological coherence between the identified TFs and the enriched pathways.

#### Dissecting TF–Target relationships by differential expression categories

We applied our comparative network analysis framework to astrocyte TF regulatory networks inferred from snRNA-seq profiles of opioid-exposed and control human brain samples. A total of 25 TFs exhibited significant differences in regulatory activity between groups, as detected by our global and local statistical tests.

To contextualize these regulatory shifts relative to gene expression, each TF–TG relationship was classified into four categories based on DE status: *Both_DE* (both TF and TG DE), *TF_DE* (TF DE only), *TG_DE* (TG DE only), and *Neither_DE* (neither DE). Across all significant TFs, we observed 37 TGs (16%) in the *Both_DE* category, 51 TGs (22%) in *TF_DE*, 66 TGs (29%) in *TG_DE*, and 91 TGs (40%) in *Neither_DE*.

When stratified by TF expression status, DE TFs were associated with 88 targets spanning Both_DE and TF_DE categories. Notably, non-DE TFs accounted for a substantial number of TG changes, with 66 TGs falling into the TG_DE category, suggesting regulatory influences decoupled from TF mRNA abundance. Overall, 29% of dysregulated targets were TG DE without TF DE, and 40% of TF–TG links fell in the Neither_DE category.

**Figure 5.**
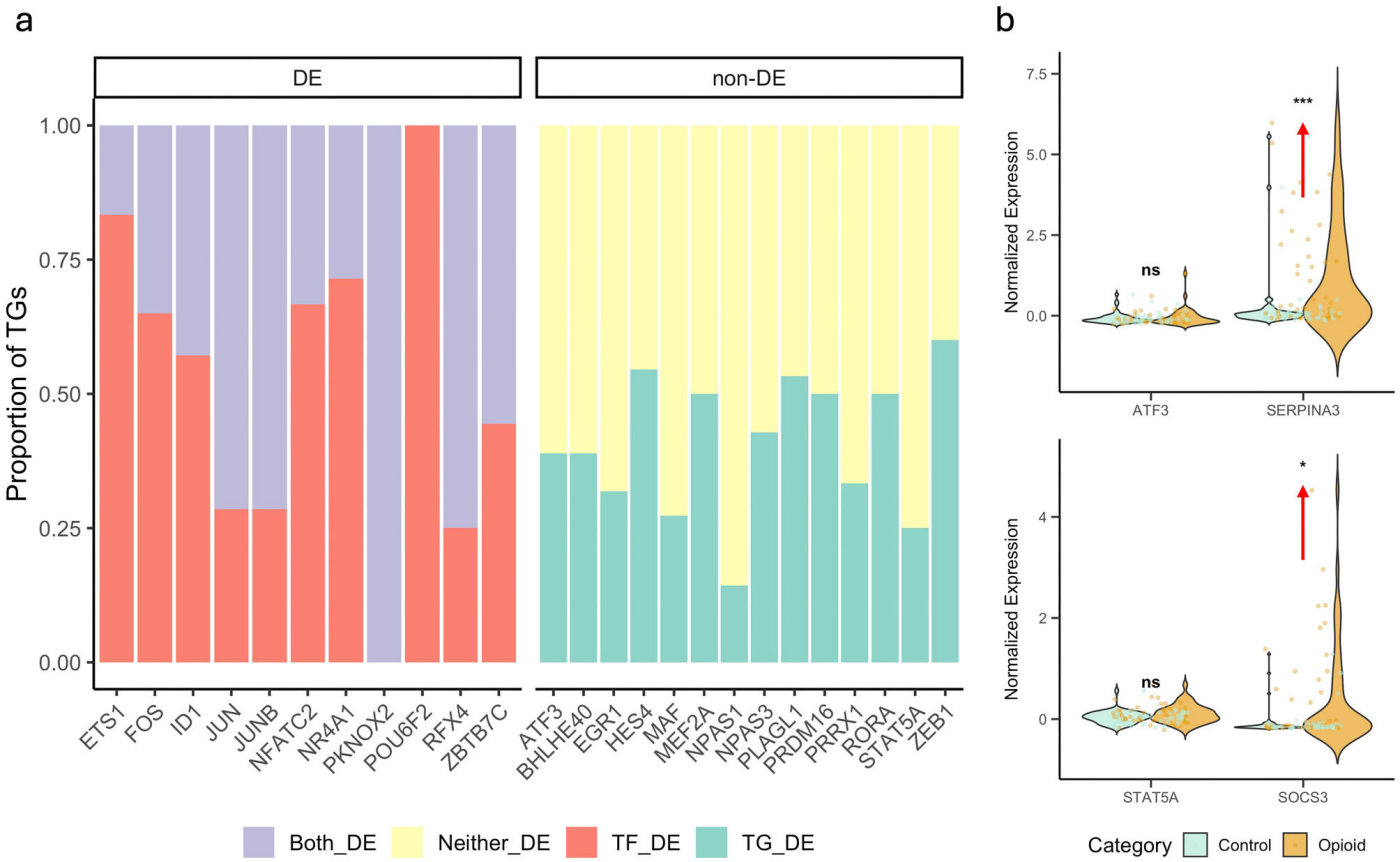
TF–TG DE relationship patterns across significant TFs. (**a**) Proportion of TGs assigned to each DE category for each TF. Bars represent the fraction of TGs classified as *Both_DE* (both TF and TG DE), *TF_DE* (TF DE only), *TG_DE* (TG DE only), or *Neither_DE* (neither DE). TFs are grouped by whether the TF itself was DE or non-DE, illustrating the heterogeneity of regulatory patterns and the frequent decoupling between TF expression and regulatory activity. (**b**) Violin plots showing expression for selected TF–TG pairs (ATF3–SERPINA3 and STAT5A–SOCS3). Significance from the Mann–Whitney U test is indicated: ***$P \le 0.001$, **$P \le 0.01$, *$P \le 0.05$, and ns (not significant). Arrows indicate direction of gene expression change.

To visualize these patterns, we generated proportional bar plots stratified by TF DE status (Fig. 5a). This analysis revealed that many TFs, including non-DE regulators, displayed substantial fractions of TGs in the TG_DE and Neither_DE categories, highlighting the complex and context-specific nature of transcriptional regulation in opioid exposure.

Case studies illustrate the biological impact of non-DE TFs (Fig. 5b): for example, ATF3 (non-DE) regulates SERPINA3, which was significantly upregulated ($P < 0.001$), and STAT5A (non-DE) regulates SOCS3, which showed moderate upregulation ($P < 0.1$). These examples underscore the value of assessing TF–TG link activity rather than relying solely on TF expression changes.

## Discussion

We evaluated five complementary statistical methods for detecting group-level differences in TF-level binary regulatory matrices (GRNs). Our framework integrates three global approaches—a U–statistics-based test (Global_U) that compares within- and between-group dissimilarity using a Hamming kernel [15, 16, 20], a distance-based pseudo-$F$ test (Global_F) adapted from PERMANOVA [18, 21], and a PCA-based test—with two local or feature-level methods: a per-feature U–test (Local_U) and Fisher’s exact test [10, 22, 23]. Through extensive simulations designed to capture realistic noise levels, signal sparsity, and network structure, we found that global methods consistently outperformed local tests in both statistical power and biological recovery, particularly when network disruptions were coordinated across many targets. Applying this framework to astrocytes from opioid-exposed and control human brain samples—demonstrated that global tests identified regulators with greater sensitivity and specificity.

The enrichment analysis revealed three major functional categories with clear biological relevance to opioid-associated astrocyte dysfunction. Neuroinflammation amplification is suggested by activation of JUN and FOS (AP-1 complex), which drive pro-inflammatory cytokines such as IL1B and TNF, potentially creating a feedforward loop that sustains chronic glial activation [28–30]. Altered astrocyte immune crosstalk is evident in STAT5A, a non-DE TF, which showed disrupted regulation of SOCS3 (TG_DE), indicating perturbation of the JAK–STAT feedback loop and sustained immune signaling [31–33]. ECM remodeling, mediated by EGR1-regulated collagen genes (e.g. COL4A1, COL6A2), may facilitate immune cell infiltration, a process relevant to opioid-associated neuroinfections [34–36].

Beyond methodological benchmarking, our analysis of the opioid dataset revealed heterogeneous expression patterns among TGs of significant TFs. We categorized TF–TG relationships into four biologically meaningful classes: (i) both TF and TG are DE, (ii) TF is DE but TG is not, (iii) TG is DE but TF is not, and (iv) neither TF nor TG is DE. This classification illustrates that TF regulatory activity can be decoupled from TF expression itself, a phenomenon observed in prior studies [37, 38]. For example, TFs with unchanged expression levels may exert altered regulatory influence through post–translational modifications or chromatin accessibility changes, leading to TG–specific expression shifts, while DE TFs may regulate TGs in buffered or context-dependent ways [39, 40]. Visualizing the distribution of these categories across significant TFs provides mechanistic insight into transcriptional control that is not apparent from DE analysis alone.

Key TF–target relationships further illustrate these mechanisms. ATF3 regulates SERPINA3 (TG_DE), a stress-responsive protease inhibitor that can potentiate neuroinflammation [41–43], while STAT5A regulates SOCS3 (TG_DE), a cytokine signaling inhibitor whose altered regulation may lead to the loss of an inflammatory brake [32, 33, 44, 45]. Notably, 29% of dysregulated targets were in the TG_DE category without accompanying TF DE (e.g. STAT5A and SOCS3), suggesting post-translational modifications such as phosphorylation or opioid-induced cofactor recruitment (e.g. $\beta$-arrestin binding). In addition, 40% of significant TF–TG links fell within the Neither_DE category, pointing to chromatin priming without transcriptional changes or compensatory regulation in homeostatic pathways [39, 40]. These findings provide a deeper layer of regulatory insight beyond conventional DE analysis.

Several actionable targets emerge from these results. The JUN/AP-1 complex is a central hub across multiple immune pathways and could be targeted with FosB inhibitors such as decoy oligonucleotides [46, 47]. The EGR1–ICAM1 axis may also represent a therapeutic avenue to mitigate neuroinflammation, as suggested by preclinical anti-ICAM1 interventions [48, 49]. Important questions remain, such as whether opioid-induced ECM changes (e.g. COL4A1, FN1) facilitate HIV neuroinvasion in individuals with OUD, and whether STAT5A dysregulation reflects a TF-specific effect or a broader disruption of IL-6/STAT3 signaling. Addressing these questions through targeted experimental validation will be critical to translate these regulatory insights into therapeutic strategies.

Beyond the biological implications, several methodological considerations merit brief mention. Computational scalability is not a current concern, as our framework operates at the sample level and present-day single-nucleus datasets rarely include extremely large numbers of samples. Nonetheless, future population-scale studies could benefit from additional benchmarking and optimizations such as parallelized permutations or feature pre-filtering.

We also note that the global U-statistic currently applies equal weights to all features. Future studies could explore biologically informed weighting schemes—based on variability, network centrality, or prior functional relevance—to further enhance sensitivity.

The PCA-based test also exhibited specificity limitations. While it performed well under coordinated signals, it showed reduced robustness in normal, balanced or noisy contexts, likely due to PCA’s reliance on dominant variance structures that may not capture localized regulatory disruptions. Within our framework, the PCA-based test should therefore be considered complementary rather than a primary driver of discovery, and future work could explore alternative dimensionality-reduction methods tailored for sparse network signals.

A limitation of the proposed framework is its reliance on binary representations of GRNs, which necessarily abstract regulatory interactions into active or inactive states. This binarization discards information about interaction strength and may obscure biologically relevant thresholds in regulatory magnitude. However, binary GRNs are a common output of current single-cell network inference pipelines and offer robustness to noise and scale differences across samples. Our results therefore emphasize detecting coordinated structural changes in regulatory programs rather than modeling graded regulatory effects. Extending the framework to accommodate weighted or probabilistic networks represents an important direction for future work.

Finally, this study remains computational in nature and lacks direct experimental validation. Although we identified TFs with altered regulatory links, confirming these findings in biological systems will be essential. Spatial transcriptomics could verify immune–astrocyte interactions in brain regions such as the nucleus accumbens, phospho-protein assays could measure activation states of TFs like STAT5A and JUN, and CRISPR interference could knock down EGR1 in iPSC-derived astrocytes to assess downstream effects on ICAM1 and immune cell adhesion. Integrating such experimental validation will strengthen the translational relevance of the framework and guide therapeutic development.

In summary, this work provides a generalizable statistical toolkit for differential analysis of TF-level binary regulatory matrices. Our integrative approach, validated through simulation and applied to opioid-associated transcriptional alterations in astrocytes, offers a foundation for further methodological and translational developments in network-based single-cell analysis.

## Supplementary Material

lqag031_Supplemental_File

## Data Availability

All analysis scripts are available at https://github.com/jianlan0816/U-test-based-GRN and https://doi.org/10.5281/zenodo.17915951. The snRNA-seq dataset analyzed in this study was obtained from the NCBI Gene Expression Omnibus (GEO) under accession number GSE240457.
