# Supplementary material for: Differential gene regulatory network analysis reveals transcriptional disruption in opioid
Source: NAR Genom Bioinform. 2026 Mar 27;8(2):lqag031. doi: 10.1093/nargab/lqag031 (PMC13126120; doi:10.1093/nargab/lqag031)
Supplement: lqag031_Supplemental_File [file lqag031_supplemental_file.pdf]

Supplementary Materials

Sensitivity of TF-Level Discoveries to FDR Threshold

To assess the robustness of TF-level discoveries to the choice of significance threshold, we repeated the TF-level analysis using a more stringent Benjamini–Hochberg adjusted cutoff of 0.05 in addition to the primary cutoff of 0.1 used in the main text. As shown in Supplementary Table S1, the majority of TFs identified at FDR ≤ 0.1 remained significant at FDR ≤ 0.05, indicating that the main findings are qualitatively stable to reasonable variations in the TF-level threshold.

Supplementary Table S1: Sensitivity of TF discoveries to TF-level FDR threshold. Checkmarks indicate significance under BH-adjusted cutoffs (0.10 vs 0.05). Abbreviations: GU, Global U; GF, Global F; PCA, PCA-based test; LU, Local U; FET, Fisher's exact test.

		GU		GF		PCA		LU		FET	
TF	Eff	0.10	0.05	0.10	0.05	0.10	0.05	0.10	0.05	0.10	0.05
FOS	0.313	✓	✓	✓	✓	✓	✓	✓	✓	✓	
ETS1	0.311	✓	✓	✓	✓	✓	✓	✓	✓	✓	
HES4	0.311	✓	✓	✓	✓			✓	✓	✓	
ZBTB7C	0.296	✓	✓	✓	✓	✓	✓	✓	✓	✓	
RORA	0.240	✓	✓	✓	✓			✓		✓	
PKNOX2	0.239	✓	✓	✓	✓	✓	✓	✓	✓	✓	
POU6F2	0.198	✓	✓			✓	✓				
ZEB1	0.293	✓	✓	✓	✓						
STAT5A	0.247	✓	✓	✓	✓						
BHLHE40	0.277					✓	✓				
PITX3	0.264	✓	✓			✓					
PRDM16	0.253	✓									
GLIS3	0.224	✓				✓					
JUNB	0.266	✓		✓							
PRRX1	0.228	✓		✓							
JUN	0.264	✓									
NR4A1	0.252	✓									
EGR1	0.312	✓									
MAF	0.292	✓									
MEF2A	0.287	✓									
ATF3	0.300	✓									
PLAGL1	0.298	✓									
ID1	0.256	✓									
NPAS1	0.247	✓									
NPAS3	0.240	✓									
RFX4	0.231	✓									
NFATC2	0.285	✓									

Sensitivity to the Choice of k in TF Effect Size

To assess robustness of TF effect size estimation to the choice of k, we recomputed TF-level effect sizes using $k \in \{5, 10, 15\}$ and compared results to the primary choice $k = 10$. As shown in Supplementary Table S2, TF effect size rankings were highly concordant across values of k, indicating that the main conclusions are not driven by a specific choice of k.

Supplementary Table S2: Sensitivity of TF effect size rankings to the choice of k. We report Spearman correlation of TF effect sizes vs k=10 and overlap/Jaccard of the top-20 TFs.

k_compare	spearman_rho	topN	topN_overlap	topN_jaccard
10 vs 5	0.990	20	18	0.818
10 vs 15	0.997	20	19	0.905
